# Skin strata delineation in reflectance confocal microscopy images using recurrent convolutional networks with attention

**DOI:** 10.1038/s41598-021-90328-x

**Published:** 2021-06-15

**Authors:** Alican Bozkurt, Kivanc Kose, Jaume Coll-Font, Christi Alessi-Fox, Dana H. Brooks, Jennifer G. Dy, Milind Rajadhyaksha

**Affiliations:** 1 Northeastern University, Boston, MA 02115 USA; 2 Memorial Sloan Kettering Cancer Center, New York, NY 10022 USA; 3 Caliber I.D. Inc., Rochester, NY 14623 USA; 4 Present Address: Paige AI, New York, NY USA; 5 Present Address: Massachusetts General Hospital, Boston, MA USA

**Keywords:** Confocal microscopy, Biomedical engineering

## Abstract

Reflectance confocal microscopy (RCM) is an effective non-invasive tool for cancer diagnosis. However, acquiring and reading RCM images requires extensive training and experience, and novice clinicians exhibit high discordance in diagnostic accuracy. Quantitative tools to standardize image acquisition could reduce both required training and diagnostic variability. To perform diagnostic analysis, clinicians collect a set of RCM mosaics (RCM images concatenated in a raster fashion to extend the field view) at 4–5 specific layers in skin, all localized in the junction between the epidermal and dermal layers (dermal-epidermal junction, DEJ), necessitating locating that junction before mosaic acquisition. In this study, we automate DEJ localization using deep recurrent convolutional neural networks to delineate skin strata in stacks of RCM images collected at consecutive depths. Success will guide to automated and quantitative mosaic acquisition thus reducing inter operator variability and bring standardization in imaging. Testing our model against an expert labeled dataset of 504 RCM stacks, we achieved $88.07\%$ classification accuracy and nine-fold reduction in the number of anatomically impossible errors compared to the previous state-of-the-art.

## Introduction

In the last decade, non-invasive skin imaging techniques have been shown to both increase diagnostic sensitivity and specificity and, critically, reduce the number of invasive procedures (e.g. biopsies, excisions)^1–5^. However, the success of these techniques typically depends on the experience of the clinician with use of the technology. This dependence remains a barrier to acceptance and integration of these techniques into dermatology practices, impeding the widespread adoption of these effective and non-invasive tools. Recent advances in artificial intelligence (AI) and machine learning (ML) methods have enabled automated and quantitative analysis of skin imaging data^6–11^. So far, the applications of ML have mainly concentrated on diagnostic analyses of the collected images. In dermoscopy, standardized skin sub-surface imaging at the macroscopic scale using a dermatoscope (magnifying glass with polarized light illumination and detection), several researchers have reported that AI methods can achieve clinician-level diagnostic accuracy^12–14^. Macroscopic imaging techniques in dermatology (e.g. dermoscopy, clinical photography) are typically surface imaging techniques that are capable of collecting color images of skin lesions. They can image entire volume of skin lesions without the ability to visualize individual layers of cells (i.e, without depth-resolution). On the other hand, higher resolution *in vivo* microscopic imaging techniques are capable of collecting thin en-face optical sections with depth-resolution, allowing for imaging of individual cell layers within a 3D volume (from skin surface to the dermis), with cellular resolution that is not provided by macroscopic imaging techniques.

Among those higher resolution *in vivo* microscopy technologies, reflectance confocal microscopy (RCM) has begun to play a unique role in diagnostic dermatology. RCM is an optical imaging technology that enables users to non-invasively examine $3\,\upmu \text{m}$–$5\,\upmu \text{m}$-thin layers (optical sections) of skin at $0.5\,\upmu \text{m}/\mathrm{px}$–$1.0\,\upmu \text{m}/\mathrm{px}$ lateral resolution at depths up to $200\,\upmu \text{m}$, typically enough to capture the *epidermis*, papillary *dermis*, and the *dermal-epidermal junction (DEJ)* in between, which is often sufficient for initial diagnosis (i.e., to triage benign versus malignant and rule out malignancy and biopsy). In this setting, selection of the imaging level has a key diagnostic importance in reading and analysis of images. For example, imaging at DEJ is especially critical as the basal cell layer, consisting of basal cells and melanocytes, at the DEJ is the germinative layer. Basal cells and melanocytes are in a near-constant state of mitosis, due to which $\sim 80\%$ of melanocytic and non-melanocytic lesions and cancers usually originate at the DEJ^1,15–18^. Therefore, in order to detect cancers at early stages (when they are still in situ), examination of this layer has a critical importance. However, the selection of the optical section/location within this 3D volume is dependent on the skills and experience of the user for visualizing and distinguishing between the different layers of cells. Therefore, standardized image acquisition in this setting remains a challenge as the process highly relies on the image interpretation abilities of the operator.

Recent studies have demonstrated that RCM imaging is highly sensitive (90 %–100 %) and specific (70 %–90 %) for detecting skin cancers^5^. Moreover, the combination of dermoscopy and RCM has been shown to increase specificity by two-fold and thus reduce the rate of biopsy of benign lesions per detected malignancy by two-fold, relative to results reported with dermoscopy alone, leading to better patient care^3,4^. This has been proven particularly valuable for lesions which lack distinct visual features and patterns and thus cannot be easily diagnosed with dermoscopy.

**Figure 1:**
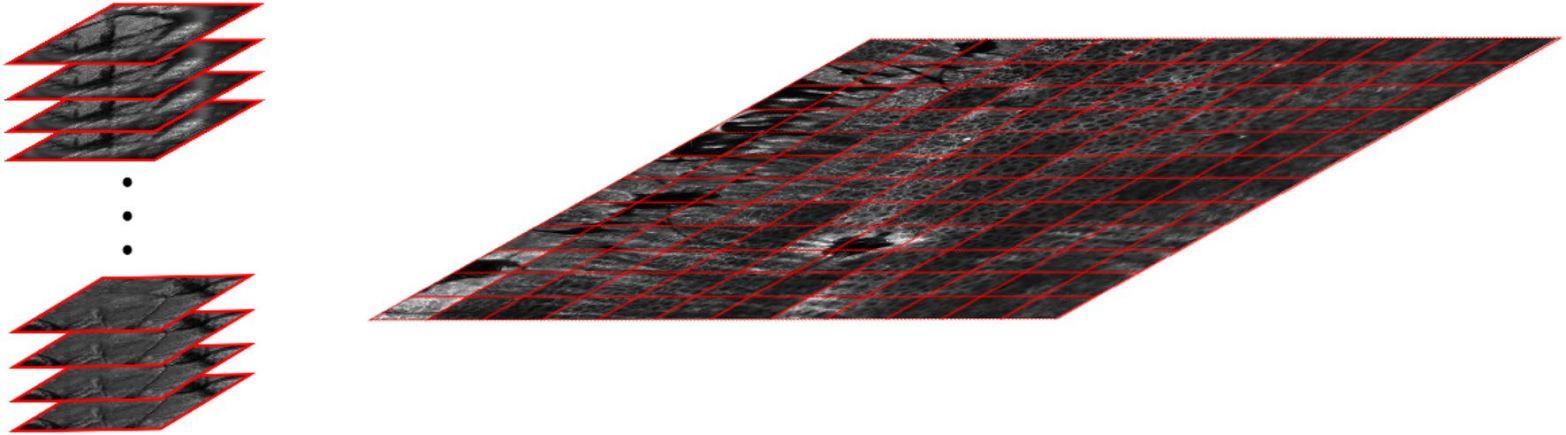
RCM Imaging modalities. (left) A 3D stack of RCM images (0.5-by-0.5 mm) used to determine the depth of different skin strata. (right) A mosaic of RCM images (6mm-by-6mm). Red borders in the mosaic represent single RCM images of the same dimensions as in the stack.

When applying RCM imaging, a single field of view (red bordered images in Fig. 1) is usually limited to $0.25\,\text{mm}^{2}$–$1.0\,\text{mm}^{2}$ with high pixel resolution in the images. However, clinicians typically must examine images over a much larger area (up to several $\text{mm}^{2}$), including the lesion and its periphery, to perform reliable diagnostic analysis. To cover the necessary area, multiple RCM images are collected at the same depth in a non-overlapping grid. These images are then tiled together to form a larger, high-resolution mosaic image. While individual RCM images can be collected in under a second, acquiring mosaics over large areas can take minutes. Diagnosis is based on the collection and reading of, typically, up to five mosaics collected at the presumed depths of the stratum spinosum including the basal cell layer, upper dermal-epidermal junction (DEJ), middle DEJ, lower DEJ, and papillary dermis. However the depth and thicknesses of each of these strata can vary not only from patient to patient, but also from site to site on the same patient, and thus the appropriate imaging depths must be accurately, consistently and rapidly identified prior to the collection of each such set of mosaics.

To do so, clinicians currently carry out a pilot imaging step first, acquiring a 3D set, or stack, of single frame (small field-of-view) RCM images at the same lateral location and separated in depth by $1.5\,\upmu \text{m}$–$5\,\upmu \text{m}$. The first of these images is acquired high in the epidermis, near the skin surface, and the last one in the deeper dermis. This set of images is referred to as an RCM *stack* and each RCM image in the stack is called an RCM *slice*. After obtaining the stack, the clinician manually classifies each slice as either epidermis, DEJ, or dermis, and then uses the stack as a reference to determine depths at which to collect *mosaics*. This process is illustrated in Fig. 1.

Following this general approach, the process of applying RCM imaging to perform diagnosis can be broken down into two steps:

1. Collecting and examining stacks of RCM images and identifying the depth of different skin strata at the location of interest on patients.
2. Collecting mosaics of RCM images at a number of diagnostically relevant depths and analyzing morphological and cellular features and patterns.

In this paper, we address the problem of automating the first step, in order to standardize and accelerate the collection of clinically relevant images for the second step.

The need for automation is particularly acute because one of the barriers to wider clinical adoption of RCM imaging is the required training for reading and interpreting the images, combined with operator dependence in terms of accurate and consistent selection of appropriate depths when collecting mosaics. These barriers occur because RCM images are visually different from histology, the gold standard diagnostic technique that the clinicians are traditionally trained for. Even if the RCM images have resolution comparable to histological images, they lack the specificity of nuclear- and cellular-level contrast provided by the exogenous dyes (hematoxlyin and eosin) used in histology. RCM images are produced by backscattered light (i.e. featuring contrast in reflectance only) and are visualized in gray-scale. The reflectance contrast is due to systematic differences in the refractive index of nuclear, cellular cytoplasmic and extra-cellular connective tissue morphological components, which results in texture differences in the images. Ideally in a capture, store, and forward implementation, where an operator acquires the images and forwards them to a clinician for diagnostic analysis, the operator must be able to analyze the texture of cellular and morphological structures in real-time at the bedside to determine the right depths for imaging^19^. However, currently, operators often exhibit highly variable accuracy in interpreting RCM images and training to read and interpret images and gain the necessary experience to accurately and consistently sample and collect images require time and effort. Automating the image acquisition process would address this barrier.

Indeed automated delineation of skin strata in RCM stacks has been a topic of interest for many researchers^20–27^. While a variety of algorithms have been applied to tackle this task, the different approaches fall into two main methods. The first, more complex, method aims to find a continuous 3D boundary between the layers of skin. The depth of different skin strata varies significantly across the skin, with the boundary between the epidermis and dermis (i.e. , dermal-epidermal junction) forming an undulating 3D surface similar to the appearance of hills and valleys. Modeling this 3D boundary can provide clinicians with a detailed understanding of how the skin varies beneath the surface, but at the cost of significant modeling difficulty.

The second set of methods approach the problem from an image classification perspective. These methods start from the premise that locating the entire 3D boundary surface is unnecessary when estimating depths for image acquisition. Instead they attempt to classify entire images from an RCM stack as epidermis, dermis, or DEJ, and then take the start and end points of each layer of skin as the points where the classifications transition between layers. The method reported on in this work falls into this second category; we perform image-wise classification of RCM slices to learn the locations of the different layers of skin. For the sake of completeness, we briefly discuss work from both categories in what follows.

In^23^, Somoza et al. use Leung-Malik (LM) filter bank based texton features^28^ to model the textural appearance of individual RCM slices. For each image in their training set, they extracted texton features, found a bag of words representation, and finally described each image as a histogram of its texton features. New samples were classified using a k-nearest neighbor classifier. Testing on image-wise labeled RCM stacks, they reported correlation coefficients of 0.84 to 0.95 between their predictions and the ground truth. Hames et al. ^24^ took a similar approach, but learned a texton representation from random 7-by-7 patches extracted from a set of training images instead of using predefined texton filters. The authors described RCM images by finding a bag of words representation of their texton filters followed by a histogram binning method. They then trained a logistic regression classifier on 235 RCM stacks of healthy skin. Their model achieved 85.6 % classification accuracy on a test set of 100 RCM stacks. Kaur et al. ^25^ leveraged the same texton extraction as^23^, taking texton filters with a support of 5-by-5 pixels. They then constructed a texton dictionary by clustering the filter outputs of randomly selected patches into 50 clusters using k-means, and used the cluster centers to form a bag of features representation. They assigned each pixel to 8 of its closest textons with a weight inversely proportional to the distance between the texton. The individual assignments for each pixel in an RCM image were binned into a histogram that they used to describe the image. The authors then trained a 3-layer neural network using these histograms. On their dataset of 15 stacks, they reported 81.73 % accuracy classifying images from the exterior epidermis, stratum corneum, stratum granulosum, stratum spinosum, stratum basale, and the papillary dermis.

Our solution, which we report in this paper, is to first train a deep convolutional neural network (CNN) to classify individual RCM images as epidermis, DEJ, or dermis and then to exploit the sequential structure of skin layers by augmenting the CNN with recurrent neural network (RNN) layers. Through the use of the recursive neural networks and the proposed attention mechanism, we take the 3D volumetric information of the skin into the account. The contributions of our work are as follows.

1. We designed a novel deep learning based classifier that can distinguish between RCM slices coming from epidermis, DEJ and dermis levels of the skin. Starting with a baseline model that is widely used for generic image classification tasks (e.g. classification of everyday images of objects and animals), we tailored it step by step according to the typical correlations within RCM stacks and the needs of RCM slice classification.
2. We compared our method to the other machine learning models that have been published for RCM strata delineation and report that we achieve significant improvement over the previous state-of-the-art results.
3. In addition to increased classification accuracy, our method also eliminated anatomically impossible transitions between skin layers that were reported by previous state-of-the-art methods by imposing the intrinsic depth-ordering of skin layers.
4. We evaluated our method on the largest dataset available for this task, 21,412 expert-labeled RCM images from 504 different stacks collected at 5 different institutes. This dataset is also notable for containing both benign and suspicious (e.g. benign nevus and melanoma) samples. To the best of our knowledge, all other datasets used for this task consisted of only healthy skin, but, of course, the primary focus and effort is on diagnostic utility for suspicious lesional skin.

Finally, we emphasize that all classifications were performed slice-wise, that is, an entire slice is classified as belonging to a single layer. This is in contrast to some of our group’s earlier work^20,22^ which belongs to the first set of methods that find a continuous 3D boundary of the skin layers. We adopted the current approach because it is more relevant to the driving clinical need to select a depth at which to subsequently acquire mosaics. However in reality a slice that is near the boundary between strata is likely to contain a mixture of regions from two distinct strata. Our “ground truth” labelers (see next section) made judgements about the dominant stratum in any give slice, and we held our classifiers to that same standard.

**Table 1:** Accuracy, sensitivity, and specificity for each method, organized by size of input each methods takes.

Input	Method	Accuracy	Sensitivity			Specificity		
			Epidermis	DEJ	Dermis	Epidermis	DEJ	Dermis
Whole stack	Toeplitz Att. ($\text{D}=1$)	**88.07**	**93.41**	**85.04**	82.66	96.35	**89.71**	96.07
	Toeplitz Att. ($\text{D}=7$)	87.59	92.84	84.18	82.89	95.87	89.38	**96.08**
	Toeplitz Att. ($\text{D}=0$)	87.57	91.63	83.97	**84.94**	**96.89**	89.40	95.29
	Toeplitz Att. ($\text{D}=3$)	87.13	91.65	83.48	83.89	96.08	89.23	95.44
	Toeplitz Att. ($\text{D}=2$)	86.65	91.44	82.27	83.95	96.08	88.91	95.07
	Toeplitz Att. ($\text{D}=5$)	86.63	91.94	83.06	81.87	95.79	88.84	95.35
	Global Att.	86.27	92.95	81.98	80.43	95.30	88.55	95.48
	Full-Seq. (Bidir. GRU)^29^	87.97	**93.95**	83.22	84.16	95.82	**90.54**	95.51
Partial stack	Partial-Seq. (Bidir. GRU)^29^	**87.52**	**94.14**	82.54	**83.33**	94.78	**90.83**	95.44
	IV3-Context^29^	86.95	92.00	**83.52**	82.64	95.61	89.24	**95.56**
Single image	Inception-V3^30^	**84.87**	88.83	**84.66**	78.18	**95.84**	85.73	**96.23**
	Hames et al.^24^	84.48	88.87	80.93	**81.85**	93.81	**87.81**	94.78
	Kaur et al.^25^	72.12	82.44	62.75	66.09	89.48	79.35	89.18

## Results

We compared a total of 13 different methods, described in detail in the Methods section. Two were methods from the literature based on hand-engineered texture features. The others all involved different deep neural networks (DNNs). These networks differed in the basic DNN structure (e.g. convolutional, residual, recurrent), in whether they included an attention mechanism^31,32^ or not, in whether they included recurrent neural network (RNN) components (e.g. gated recurrent units^33^ (GRUs)) or not, and in whether they used only the single slice of interest, a local neighbourhood of the slice, or the entire stack. We report test set performance for all of those models in Table 1. The table is broken into three blocks of rows, where all models in the same block share the same type of input. The first block consists of our attention based models. The models reported on in this block take in entire RCM stacks and output predictions for each image in the stack. Within this block, Toeplitz Attention models are further distinguished by a parameter we denote *D*, which is the support of the attention weights in one direction (i.e. length of the attention vector is $2D+1$). The second block contains models that take in partial-sequences to make predictions for individual RCM images. This includes our partial-sequence RCN models, as well as an Inception-V3 model that we trained using the partial-sequence data (IV3-Context in Table 1) as a baseline. The last block contains models that classify single RCM slices, including Inception-V3^30^ that we trained as baseline, and other models proposed in the literature^24,25^. Within each block the methods are sorted in descending order of test accuracy. In addition to test accuracy, we also report sensitivity and specificity for each of the three classes. The highest accuracy, specificity, and sensitivity for each row block of models are marked in bold.

### Error analysis: boundary errors vs anatomically inconsistent transitions

The main goal of incorporating the sequential nature of RCM stack data into our model was to build a classifier that can leverage the monotonic structure of skin structure to increase classification accuracy. In this subsection, we analyze the types of errors made by each model to understand how well they learned these constraints. To do so, we categorize classification errors into two different types. One type of error is inaccuracy; it occurs when a boundary starts shallower or deeper than its actual location in a stack. As noted earlier, slices acquired near boundaries are expected to contain features from both layers (classes). Therefore, small boundary location errors of this type are inevitable. The second type of error, which we refer to as *inconsistency* errors, are detected strata transitions that violate the sequential constraints of the skin. These physiologically inconsistent transitions. when moving down from surface to dermis, are epidermis$\rightarrow$dermis, DEJ$\rightarrow$epidermis, dermis$\rightarrow$epidermis, dermis$\rightarrow$ DEJ. To quantify the consistency of a model, we counted the number of these physiologically inconsistent errors and report these numbers for the best full-sequence and partial-sequence RCNs, the Inception-V3 model, and the models presented in^24^, and^25^ in Table 2. We want to emphasize that these results do not directly correspond to accuracy; it is possible for a set of labels to be consistent but not accurate.

**Table 2:** Number of anatomically inconsistent predictions made by each model. Models are sorted in ascending order with respect to the total number of errors.

Method	Error types				Total
	Epidermis $\rightarrow$ Dermis	DEJ$\rightarrow$ Epidermis	Dermis$\rightarrow$ Epidermis	Dermis $\rightarrow$ DEJ	
Global Att.	0	0	0	0	0
Toeplitz Att. ($\text{D}=1$)	1	1	0	1	3
Toeplitz Att. ($\text{D}=5$)	0	1	3	0	4
Toeplitz Att. ($\text{D}=7$)	0	5	1	1	7
Full-Seq. RCN^29^	0	4	0	3	7
Toeplitz Att. ($\text{D}=3$)	0	7	2	0	9
Toeplitz Att. ($\text{D}=0$)	0	18	0	0	18
Toeplitz Att. ($\text{D}=2$)	0	22	0	0	22
Inception-v3^29^	3	25	8	32	68
Hames et al. ^24^	14	59	11	56	140
Kaur et al. ^25^	12	133	12	44	201

### Delineating the epidermis-DEJ and DEJ-dermis boundaries

As our ultimate goal is to delineate the epidermis-DEJ and DEJ-dermis borders, we also quantify the performance of our RCNs by looking at the distribution of the distance between the predicted boundaries and the ground truth boundaries to the classifications from the methods in Table 2. To obtain consistent transition boundaries, we use a two-step post-processing heuristic. In the first step we applied a 3-layer median filter to remove outlier classifications. In the second step, we then applied a causal max filter, which replaces each prediction with the maximum value in the sequence of predictions before it.

**Table 3:** Mean absolute error (MAE) of models for estimating epidermis-DEJ and DEJ-dermis boundaries. Models are sorted according to epidermis-DEJ boundary estimation MAE.

Model	Mean absolute error ($\upmu \text{m}$)	
	Epidermis-DEJ Boundary	DEJ-Dermis Boundary
Toeplitz Att. ($\text{D}=1$)	5.76	9.24
Toeplitz Att. ($\text{D}=7$)	6.79	8.87
Full-Seq. RCN	6.86	9.29
Toeplitz Att. ($\text{D}=0$)	6.94	9.34
Toeplitz Att. ($\text{D}=5$)	7.16	9.71
Global Att.	7.23	10.65
Toeplitz Att. ($\text{D}=2$)	7.47	9.70
Toeplitz Att. ($\text{D}=3$)	7.83	9.10
Inception-v3^30^	9.82	10.59
Hames et al. ^24^	11.40	11.72
Kaur et al. ^25^	18.20	19.45

**Figure 2:**
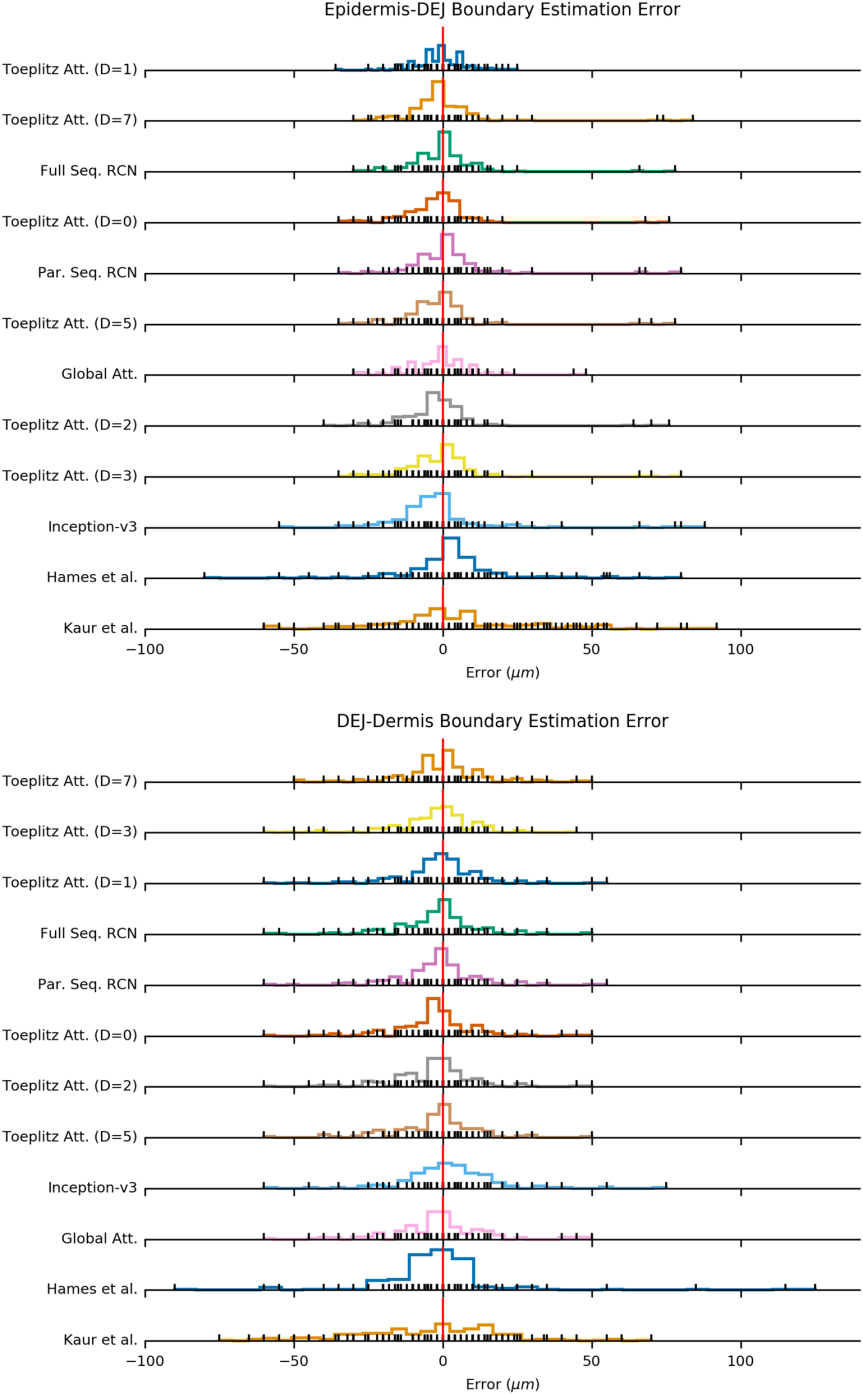
Histograms of error for the predictions of the epidermis-DEJ and DEJ-dermis boundaries for each model. Each black tick indicates a value of error that occurred in a test stack, colored lines show the distribution of errors, and red vertical line shows the origin. Models are sorted according to mean absolute error.

## Discussion

There are a number of interesting observations that we can make based on the results in Table 1. The best model overall was the Toeplitz Attention ($\text{D}=1$), which achieved 88.07 % overall accuracy on the test set. This model outperformed the best previously published method^24^ by 3.59 %, representing a 23.13 % reduction in classification error rate. Comparing both the full-sequence and partial-sequence models, we observe that unidirectional GRU and both standard RNN models trained using the full-sequence scheme were outperformed by nearly all bidirectional methods, the one exception being the partial-sequence unidirectional GRU model. In the full-sequence training scheme, we processed the entire RCM stack at once and relied on the network’s ability to identify the important information for classifying each image, whereas in the partial-sequence scheme, we effectively predetermine that the neighboring images contain the relevant information necessary to classify an RCM slice. We hypothesize that the simpler unidirectional and standard RNN architectures had a more difficult time learning in the full-sequence scheme. Following this logic, it is reasonable to conclude that RCM images beyond the immediate area of the target slice contain some important information for classification of that slice, and that the more complex bidirectional GRU network was able to leverage this information to increase classification accuracy.

On the other hand, in the case of attention models, where the model can also learn how to weight neighborhood information into the final decision, we observed benefits of using larger neighborhood in certain cases. Table 2 shows that using global attention helped in eliminating all the anatomically impossible predictions. Moreover, as shown in Table 3 and Fig. 2, using an attention model with a larger neighborhood resulted in increased precision in localization of DEJ-Dermis boundary. We suspect that the main reason for this result is that, due to loss of resolution and contrast, the DEJ-Dermis boundary is more ambiguous and harder to find compared to the Epidermis-DEJ boundary. Therefore, the model more effectively utilizes the information from a slightly larger neighborhood to delineate the DEJ-Dermis boundary.

Sensitivity and specificity were very similar across our experiments and appeared to vary proportionally with test accuracy. However, it is interesting to note that all of our models were less sensitive to the DEJ and dermis. This is consistent with other results in the literature^21^. A typical stack of RCM images will contain more epidermis than DEJ and dermis samples because reflectance confocal microscopes can only image down to the papillary dermis, (44 % of samples in our dataset are epidermis, compared to 34 % DEJ and 22 % dermis). Moreover, due to optical aberrations that start below the DEJ and worsen around the deeper rete ridges (valleys of the undulating DEJ boundary), the DEJ-to-dermis boundary usually appears fuzzy, making it harder to detect. Thus, the level of DEJ-to-dermis boundary in a given stack is partially subjective, even for expert readers. This uncertainty helps to explain the lower sensitivity to DEJ and dermis compared to epidermis.

While our recurrent models perform better compared to state of the art models, it is worth noting that the logistic regression model presented by Hames et al. achieved performance comparable with the Inception-V3 network. While the recurrent models provide significant improvements in testing accuracy, they lack the interpretability of the regression approach which is a potential drawback in medical applications.

Analyzing the types of the errors that each model lead to, as presented in Table 2, methods that do not take full stack information into account produce more inconsistencies. The partial-sequence RCN produces ${\sim }3\times$ fewer inconsistencies than the best single-image model even though it only takes into account neighborhoods of three images. The full-sequence RCN performs significantly better than the other methods, with ${\sim } 3\times$ fewer inconsistencies than the partial-sequence RCN and thus ${\sim } 9\times$ fewer inconsistencies than the best non-RCN model.

Analyzing the Epidermis-DEJ boundary error distributions in Fig. 2 and Table 3, we see the full-sequence RCN, and partial-sequence RCN achieved the lowest median error ($5\,\upmu \text{m}$). The method proposed in^24^ and the Inception-V3 model follow with a median error ($6\,\upmu \text{m}$). Even if this difference is not statistically significant, our methods lead to fewer physically inconsistent errors (Table 2) compared to the other methods in the absence of any post-processing heuristics described in section. Therefore, for a fairer comparison, Fig. 2 should be analyzed together with Table 2, which shows the number of errors the heuristic algorithm needs to correct. A similar result follows for the DEJ-dermis boundary.

## Methods

### Dataset

The dataset used in this work is composed of 504 RCM stacks that were gathered from two different studies across six different clinical sites. 196 out of the 504 stacks were collected at Memorial Sloan Kettering Cancer Center (New York, NY, and Hauppauge, NY), the University of Rochester (Rochester, NY), Loma Linda University Health (Loma Linda, CA), and Skin Cancer Associates (Plantation, FL) under a study approved by Institutional Review Boards (IRB) of each institution. Informed consent was obtained from all the subjects participating the study. The other 308 stacks were from a study conducted by the Dermatology Research Centre at the University of Queensland (Brisbane, Australia)^24^, and publicly available at the Dryad repository (https://datadryad.org/stash/dataset/doi:10.5061/dryad.rg58m). All the data is de-identified (patient metadata was removed). All experiments were performed in accordance with relevant guidelines and regulations.

The overall dataset consists of 21412 RCM images. All images in all stacks were acquired with $0.5\,\upmu \text{m}$ lateral resolution and $3\,\upmu \text{m}$ optical sectioning. The dataset contains normal, benign melanocytic, and diseased skin samples of the arms, the legs, and the torso. This is critical, as noted, since clinicians typically image suspicious lesional skin whose appearance is very different from healthy and/or non-lesional skin. Each individual image in all the stacks was labeled by at least two experts as belonging to one of four classes: stratum corneum, epidermis, DEJ, or dermis. For this study, we merged the stratum corneum (the topmost layer of the epidermis) and the epidermis classes together and then we carried out a 3-way classification. For our experiments, we partitioned the dataset into training, validation, and testing sets of 245, 61, and 198 stacks respectively. To handle the case where one patient may have multiple stacks in the dataset, we stratified the partition patient-wise (i.e. all stacks from a particular patient are exclusively in the training, validation, or testing set).

### Recurrent convolutional networks

Human skin maintains a strict ordering of different strata; the transition between the layers are contiguous, non-repeating, e.g. epidermis$\rightarrow$ dermis$\rightarrow$ epidermis transitions are not possible) and monotonic (dermis$\rightarrow$ DEJ or DEJ$\rightarrow$ epidermis transitions are also not possible). These constraints provide powerful cues that are exploited by human experts for more accurate classification. Given this sequential structure, recurrent neural networks naturally lend themselves to the problem of skin strata identification, as they are able to take the sequential dependencies between different images in a stack into account.

Within each RCM image, there is also a significant amount of spatial information present in the varying texture of the tissue. In previously reported work, convolutional neural networks have demonstrated the ability to learn high-level features from images, and have been applied with great success to numerous image classification tasks^34–36^.

Given these characteristics of our data, we adopted a hybrid neural network architecture with both convolutional and recurrent layers similar to that proposed in^37^. We first trained a deep convolutional network to learn important spatial features for the classification of individual RCM images, and then augmented the network with recurrent layers so that the classifier could account for the features of other RCM slices. Following the convention used in^37^, we refer to models with this structure as recurrent convolutional networks (RCNs). For our deep CNN architecture, we used a modified Inception-V3 model^30^, where we added an additional fully connected layer with 256 neurons before the last layer.

After training the model to classify individual RCM images as epidermis, DEJ, or dermis, we removed the 3-class classifier layer and the non-linearity on the penultimate fully connected layer. We then fixed the weights of the trained network and appended recurrent layers. The two different techniques that we experimented with for training the recurrent layers are explained in the following sections.

#### RCNs without attention

**Figure 3:**
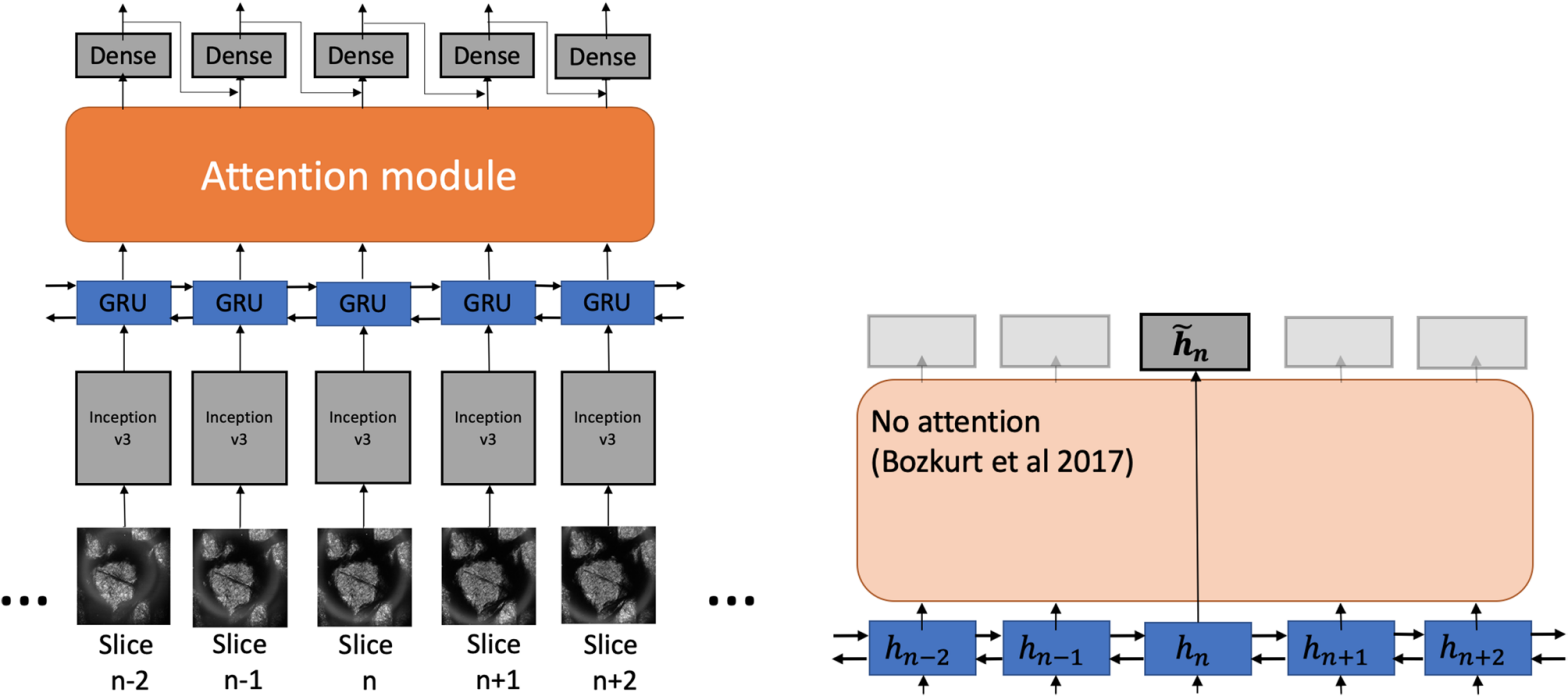
(left) Attention module allows fusion of GRU embeddings of different slices. (right) When no attention is applied ($\tilde{h}_n=h_n$) model is equivalent to full-sequence RCN^29^.

The first approach was training the RCN model on sub-sequences containing a local neighborhood (in depth) of *N* images around the subject RCM image. For every sample in our dataset, we constructed a sequence of *N* RCM slices centered around the target sample. We then trained our network on batches of these sub-sequences. This training procedure imitates the technique of examining neighboring RCM slices that dermatologists apply while classifying RCM images. We experimented with two different scenarios. In one, a model was trained using a neighborhood of three slices ($N=3$), trying to estimate the label of the middle slice. The second scenario involved training the RCN model using the full stack. As illustrated in Fig. 3, the model processes the entire RCM stacks (full-sequence) and outputs predictions for each image in the stack. This approach is potentially more flexible, as we provide the model with the complete RCM stack and allow it to learn the information that is useful for slice-wise classification.

#### RCNs with global attention

The main disadvantage of the partial sequence approach is the need for the user to set *D*, the size of the neighborhood, during training time; once set, it cannot be changed. Our initial experiments show that our CNN-based model was typically more confident in its decisions for the RCM slices from superficial epidermis and deeper dermis, where the structures are more distinct. On the other hand, around the DEJ, the decisions became less confident. Therefore, a model that adaptively changes the neighborhood size according to depth might be ideal. In response to this observation, we implemented an attention mechanism on top of the full sequence scenario, so that the model could determine the neighborhood size on-the-go during processing.

**Figure 4:**
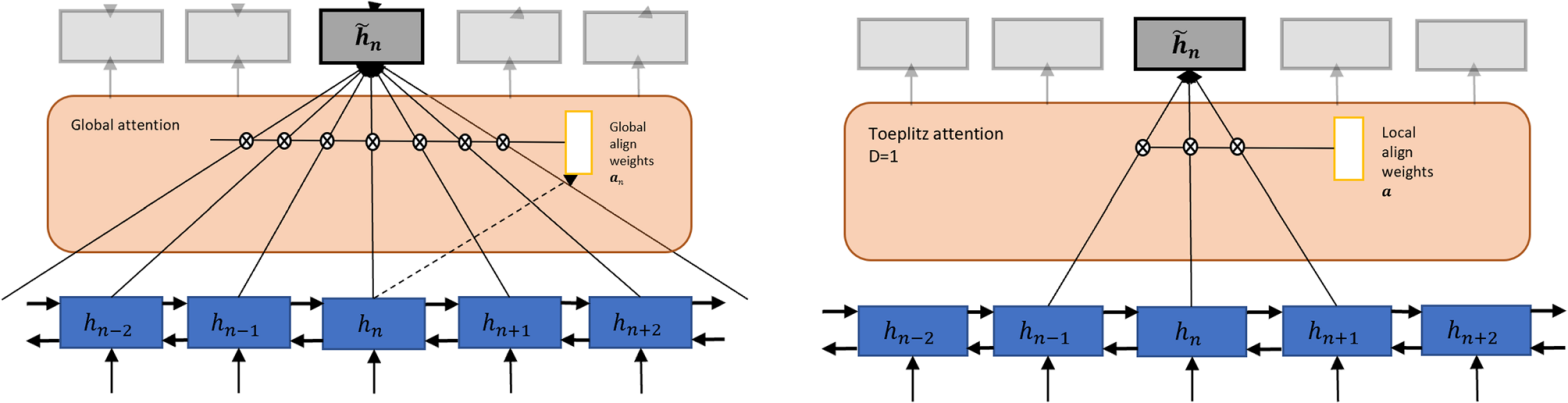
Attention mechanisms: (left) In the global attention model output decision for a particular slice is affected by all the slice in the stack, whereas (right) in the Toeplitz attention model the output is only affected by a local neighborhood of slices (e.g D = 1 in the exemplar). Note that this figure is intended to explain only the attention layers, the encoder and decoder structures can be seen in Fig. 3.

Global attention^31^ has been proposed as a way to align source and target segments in neural machine translation in a differentiable manner. It has been used in many computer vision and natural language processing tasks^38,39^. Specifically, for each depth *n*, an attention vector $\mathbf{a}_n$ with the same length as the sequence $\{\mathbf{h}_0,\dots , \mathbf{h}_N\}$ is calculated with by a multi-layer perceptron from $\mathbf{h}_n$, the encoding of the $n$th slide. Then a context vector $\tilde{\mathbf{h}}_n$ is calculated as weighted sum of encodings with weights set according to the attention vector (Fig. 4, left).

#### RCNs with Toeplitz attention

Ideally, the network should imitate the dermatologist in looking at only a few slides per classification. This translates to $\mathbf{a}_n$ being sparse, i.e. elements of $\mathbf{a}_n$ should be non-zero for only a few slides. This is known as “hard attention”^40^, and is hard to train, due to the mechanism not being end-to-end differentiable.

Here, we propose a simplified attention model, named Toeplitz attention, to overcome this issue. The name Toeplitz attention comes from the idea that the attention map created by this method has a Toeplitz structure, that is to say that weights are constant with respect to offset from the slice being estimated – the value of the weight depends only on that offset and not on the global location of the slice in the stack. Support of the attention weights is more compact than it is in global attention, but the network is still end-to-end differentiable, therefore easier to train than hard attention.

This mechanism can be seen as a special case of local attention with monotonic alignment^32^, where the context vector $\tilde{\mathbf{h}}_n$ was calculated as a weighted average over sets of $\mathbf{h}_n$ within a window $n' \in [n-D, n+D]$ (*D* is chosen, in both^32^ and our work, empirically). Now $\mathbf{a}_n$ has a shorter support of $2D+1$, compared to the input sequence length in the global attention case. It is calculated in a similar fashion to global attention^32^. In our case, the elements of $\mathbf{a}_n$ with the window $n' \in [n-D, n+D]$ are depth-independent, i.e. $\mathbf{a}_n=\left[ \mathbf{0}_{n-D-1}, \mathbf{a}, \mathbf{0}_{N-n-D}\right]$ where $\mathbf{a}$ is a learnable kernel of length $2D+1$ with all non-negative entries that sum to one (convex combiner), and $\mathbf{0}_{n-D-1}$ is a zero vector of length $n-D-1$. The attention map *A*, which is a concatenation of $\mathbf{a}_n$ for each slice, $A^T=[{a}_1^T,\ldots ,{a}_N^T]$ therefore will have a Toeplitz structure. This structure lends itself to an efficient implementation using convolution.

In the neural machine translation literature, the attention layer is typically applied between an encoder and decoder^33,41^. We replicate this structure in our work as well. We use bidirectional gated recurrent units (GRU)^33^ appended to Inception v3 networks^30^ to create a recurrent encoder network. This network will produce an encoding for every image in a stack. The full-sequence RCN model used in^29^ can be formed by attaching a fully connected layer at the end of this encoder. From this lens, it can be seen as a special case of an attention-augmented network. Indeed, we can recover the full-sequence RCN as a special case of Toeplitz attention where $D=0$, so the attention map becomes an identity matrix.

We use different decoder networks for each attention mechanism. For global attention, we use a GRU followed by a fully connected layer. For Toeplitz attention, we use simply a fully connected layer. In both cases, we augment the attended encodings (context) with the decoder’s output (class probabilities) at the previous time step (again, time corresponds to slice depth here), to efficiently exploit the structure nature of the data.

### Experiments

All RCN models were implemented using the Keras^42^ library and trained on a single NVIDIA Tesla K40, Titan X GPU, or Titan V GPU.

The original Inception-V3 network is designed for RGB images. We modified the first layer Inception-V3 architecture to accept single-channel inputs, since RCM images are grayscale. While training the CNN, we also augmented our dataset with randomly sheared, zoomed (magnified), rotated, stretched, horizontally and vertically flipped versions of training images.

We were not able to train full RCN models end-to-end, as the batch normalization layers in inception-V3 model are not designed to be trained in a time-distributed setting. Removing these layers allowed us to train the complete RCN, but the model performed significantly worse. To overcome this problem, we first trained a CNN for image-wise classification task using the RCM images in the training set. We then removed the last layer of the trained CNN and used the remaining network as a feature extractor to obtain feature representations for each slice in the dataset. The recurrent layers are then trained on sequences of these extracted features. While this approach makes experimentation with different CNNs more difficult, it allows us to use CNNs with batch normalization, and avoids significant redundant computation while training different RNNs. It also enabled us to train full-sequence models on a single GPU, as the full CNN + RNN model was too large to fit into GPU memory.

All RCNs in Table 1 have two recurrent layers with 64-dimensional embedding (each layer corresponding to one direction), followed by a fully connected layer and a softmax for classification.

Following the same steps in^29^ for training the RCN models, we created the input data by concatenating the extracted CNN features of all slices for every stack. Because our dataset contains stacks of various lengths, we fixed the maximum sequence length to 71 slices (The single RCM stack in our dataset of length greater than 71 was clipped from 101 to 71 slices for the full-sequence models) and zero-padded shorter sequences. The padding was then masked out during training and testing. The RNN layers were trained for 200 epochs with a batch size of 4 sequences, and a learning rate of 0.001. To avoid overfitting, we used a 10% dropout rate on the recurrent connections and an L1 regularization penalty on the recurrent weights with a weighting coefficient of 0.05.

For each RCN model, the model snapshot with the best validation accuracy was selected, and its performance on the test set is reported in Table 1.

For comparison, we used the publicly available code from^24^. We also implemented the method presented in^25^ by following the instructions in their paper. We report the results of training and testing on our dataset using these methods in Table 1. We will make the code available upon publication.

## Conclusion

In this study, we presented a method based on deep convolutional and recurrent neural networks for classifying skin strata in RCM stacks. We evaluated our method on the largest and most comprehensive dataset for this task, and demonstrated a significant increase in the accuracy of skin strata delineation in RCM stacks. The test scenario used in this study is more realistic compared to those used by most previous methods, in the sense it contains significant samples of lesioned skin, not just normal skin, and clinicians are necessarily most concerned about imaging suspicious lesions rather than normal skin. In addition to increased classification accuracy, our best RCN achieved a ${\sim }9\times$ reduction in the number of anatomically inconsistent transitions between layers of skin when compared to the previous state-of-the-art methods. Our experiments show that our method outperforms techniques designed for smaller datasets that comprise only healthy skin, and other deep learning based methods which do not incorporate full stack information. Overall, our results are an example of the idea that combining knowledge of the intrinsic properties of a dataset with the strengths of deep neural networks can yield a powerful tool for solving medical imaging problems, and can help to guide clinicians in their clinical practice.
